# The Antibacterial Effects of Cocktail and Single Forms of Lytic Phages Belonging to *Podoviridae* and *Myoviridae* Families from Sewage against *Shigella sonnei* and *Shigella flexneri*

**DOI:** 10.1155/2022/7833565

**Published:** 2022-11-25

**Authors:** Javad Jokar, Niloofar Rahimian, Abdolmajid Ghasemian, Sohrab Najafipour

**Affiliations:** ^1^Department of Microbiology, Faculty of Medicine, Fasa University of Medical Sciences, Fasa, Iran; ^2^Noncommunicable Diseases Research Center, Fasa University of Medical Sciences, Fasa, Iran

## Abstract

**Background:**

Diarrhea caused by bacterial pathogens such as *Shigella* spp. is one of the prominent public health concerns. The evolution of vast antibiotic resistance by these pathogens, leading to failure in the infections eradication, has made an impetus to seek and develop novel approaches. Recently, some alternative therapies such as phage therapy have been investigated. Bacteriophages are viruses that target specific bacterial species. The objective of this study was to assess the therapeutic effect of phages obtained from hospital sewage against *Shigella sonnei* (*S. sonnei*) ATCC® 9290 and *S. flexneri* ATCC 12022 standard and clinical strains.

**Methods:**

Four various lytic bacteriophages were isolated from animal fecal and sewage samples and propagated using *S. sonnei* and *S. flexneri* as host organisms. The phages' morphology was determined using transmission electron microscopy (TEM). The lytic potential and host specificity of isolated phages were evaluated using double layer plaque assay and spot test. Moreover, bacterial turbidity values were evaluated in coculture with phages in the Luria Bertani (LB) medium for 24 hours at time intervals of 30 min.

**Results:**

Phage cocktails (Shs1, Shs2, Shf1, and Shf2) exhibited higher antimicrobial activity than single phage application against *S. sonnei* and *S. flexneri* standard strains. The phages belonged to *Podoviridae* and *Myoviridae* families according to TEM-assisted morphological features analysis. In addition, the phages exhibited host specificity using the spot test against 18 *Shigella* clinical isolates.

**Conclusion:**

In this study, phage cocktail of *Podoviridae* and *Myoviridae* families from sewage conferred substantial antibacterial effects against *S. sonnei* and *S. flexneri*. However, single phage effects were unstable in the LB coculture. Moreover, the phages had host specificity using the spot test performed against *Shigella* spp. clinical isolates.

## 1. Introduction

Among the bacterial pathogens, *Shigella* species are among the most common and leading isolates in patients with diarrhea which employ various virulence factors for severe forms of infections [1, 2]. *Shigella* is a Gram-negative pathogenic genus that causes bacillary diarrhea in humans. *Shigella* species are transmitted through oral-fecal route and enter the human body following contaminated water and food. These bacterial species have low infectious dose being 10^1^-10^2^ colony forming unit (CFU)/mL to cause infection [3]. Infection caused by this pathogen leads to severe and acute inflammatory bowel disease characterized by watery diarrhea with purulent discharge. The emergence and spread of multidrug-resistant (MDR) strains and their resulting mortality are a major concern [4, 5]. In recent years, efforts have been made to isolate and optimize phages (natural antiviral agents) to replace antibiotics [6, 7]. Phage cocktail is a mixture of several types of purified phage particles to kill all subspecies of a pathogenic bacterium. The most important feature of phages is the high efficient killing of host bacteria. Even when lower particle doses are applied, the phages still function unyielding. This is not the case with the use of chemicals, and as the dose is reduced, treatment responses decline dramatically. This is due to the so-called “auto dosing” properties of phages [8–10]. Another advantage is their narrow spectrum or host specificity of phages, each infecting a particular range of bacterial species. The phage character does not alter the normal flora of the body, maintaining health conditions [9, 10]. Loss of normal flora in the body leads to a decrease in some vitamins and impaired intestinal functions. In spite of synthetic compounds, phages exert no side effects. In previous studies, phage-based products have been employed mainly in food and limited to microbial species including *E. coli* O157:H7, *Listeria monocytogenes*, *Campylobacter*, and *Salmonella* species [8, 10–12]. The advantages of using bacteriophages as natural food preservatives include high specificity, low cost, self-limiting, and the possibility of isolation from various environmental sources. Despite the benefits mentioned, some challenges remain such as phage resistance, side effects, positive and negative phage convergence, and the possibility of virulent phage mutation and alteration to moderate (lysogenic) phage for efficient combating against pathogens [13–15]. Noticeably, the antibacterial effects of various phages environmental from environmental sources have been delineated against several bacterial pathogens such as *Klebsiella pneumonia*, *Acinetobacter baumannii*, *Staphylococcus aureus*, *E. coli*, and *Shigella* species giving admissible results [6–15]. The aim of the current survey was to assess the therapeutic effect of phages cocktail and single form obtained from hospital sewage in Southern Iran against *S. sonnei* ATCC® 9290 and *S. flexneri* ATCC 12022 standard strains.

## 2. Methods

### 2.1. Bacterial Strains

*S. sonnei* ATCC® 9290 and *S. flexneri* ATCC 12022 standard strains were obtained from Iranian National Center for Genetic and Biologic Resources. Eighteen clinical isolates were also collected for assessment of host-specific antibacterial assay. Additionally, *Staphylococcus aureus* (*S. aureus*) ATCC 43300 was employed as the control. The strains were subcultured onto the blood agar (Merck, Germany) and MacConkey (Merck, Germany, for *Shigella* species) media and kept at -20°C until further experiments. Furthermore, 18 clinical isolates of both species were collected and identified using biochemical tests.

### 2.2. Antibiotic Susceptibility Test

The antibiotic susceptibility test was implemented according to the procedure provided by clinical and laboratory standards institute (CLSI) version 2020. The disks included amoxicillin (30 *μ*g), imipenem (10 *μ*g), erythromycin (30 *μ*g), ciprofloxacin (5 *μ*g), co-amoxiclav (30 *μ*g), gentamicin (30 *μ*g), cefotaxime (30 *μ*g), ceftazidime (30 *μ*g), and tetracycline (30 *μ*g). *S. aureus* ATCC25923 and *Escherichia coli* ATCC25922 were used as control of the test quality [16]. Nonsusceptibility to at least one antibiotic in three groups was defined as the MDR as per the CLSI guidelines.

### 2.3. Isolation of Phages

Hospital sewage (Fasa Vali-Asr Hospital, south of Iran) and animal fecal samples (200 mL) were collected at different time points. The samples were stored at 4°C and processed during 48 hrs. Next, the samples were centrifuged at 5000 rpm for 5 min, and the supernatant was passed through the 0.22 *μ*M filters and used for antibacterial assays. Ten microliter of each supernatant solution was added to 99 mL of Luria Bertani (LB) medium, and also 0.1 M of each CaCl2 and MgSo4 was added. The solution was placed in a shaker (120 rpm) at 37°C for 16 hrs. Then, the solution was centrifuged at 5000 rpm at 4°C for 7 min. The supernatant was passed through the 0.22 *μ*M filter paper (PALL Acrodisc, Port Washington, NY, USA) and diluted serially [17, 18].

### 2.4. Double Layer Plaque Assay

In each sterile tube, 100 *μ*L of supernatant suspension was added and diluted 10^−1^-10^−6^ times into SM solution (5.8 g NaCl, 2 g MgSo4, 7H2O, 5 mL of 1 M Tris, and 5 mL of 2% gelatin in a total volume of 1 liter). Also, 200 *μ*L of bacterial suspension in the logarithmic phase was added to each tube, and subsequently, 3 mL of melted agarose (soft agarose, 0.7%) was added and mixed and immediately poured into agar plates. Then, the plates were incubated at 37°C for an overnight. In case of phage presence, after the multiplication in the agar medium, the plaque formation was observed.

### 2.5. Spot Assay

In this test, after mixture of 200 *μ*L of bacterial isolates with 3 mL of soft agar and pour in agar plates, 10 *μ*L of lysate suspension (10^9^ pfu/mL) was added in three spots and incubated at 37°C overnight. The positive result was confirmed following observation of no-growth zones indicating bacterial lysing property.

### 2.6. Purification of Phage

For differentiation of probable multiple phages or isolation of each phage singly from plaque or inhibition zones, the plaque assay stage was repeated in triplicate to obtain pure plaque from each phage. For phage precipitation, 5X ethylene glycol (PEG) was used. Phage suspension and PEG were mixed in a ratio 1 : 5 in a 50 mL falcon and hand-shaked gently for a minute and placed onto ice. Next, the falcons were immediately centrifuged at 4°C and 9000 rpm for 15 min. The upper part of the supernatant was removed, and centrifugation was repeated. The tube precipitate was observed, and the tube was dried. The precipitates were solved into SM solution. The fluid was taken using filter paper after 10 min. The images of phages morphologies were taken using TEM (EM10c, Zeiss) [17–19].

### 2.7. Phage Morphology Determination

The morphology of phages was determined using transmission electron microscopy (TEM). Briefly, one droplet of pure phage suspension was placed on the copper mesh covered with carbon for 5 min. Next, the phage was taken using a pipette and stained using uranil acetate (TAAB Laboratory, UK).

### 2.8. Assessment of Antibacterial Effects of Phages

Antibacterial effects of single and cocktail phages were assessed as previously described [19, 20]. Briefly, *S. sonnei* ATCC® 9290 and *S. flexneri* ATCC 12022 standard strains were cultured into the LB medium with shaking at 40 rpm at 37°C to reach logarithmic phase of growth. Next, single and cocktail phages (a ratio of 1 : 1 : 1) at 8 log PFU/mL were subjected to bacterial suspensions at MOI (multiplicity of infection) values of 10 and 5, respectively, for 24 hrs and time intervals of 30 min.

### 2.9. Host Specificity Using Spot Test

In order to evaluate the specific effects of each phage to inhibit the bacterial growth, 10 *μ*L of lysate phage (at concentration of 8 log PFU/mL) was taken and subjected to 0.5% bacterial LB agar bacterial lawn culture. Following incubation at 37°C for 18-24 h, the plaque formation and size were observed with naked eye to determine specific effect [19, 20].

### 2.10. Data Analysis

The data was analyzed using SPSS, version 21, and applying chi-square and Mann–Whitney test, in which a *p* value <0.05 was considered as a significant finding.

## 3. Result

### 3.1. Antibiotic Resistance Pattern of *S. Sonnei*

**Figure 1:**
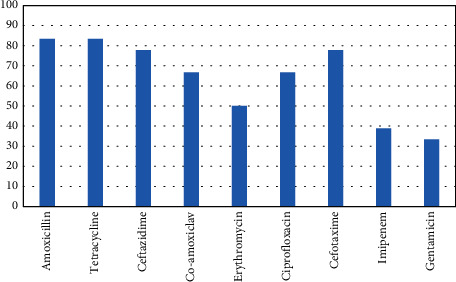
The antibiotic susceptibility test.

Of 18 *S. sonnei* isolates, the majority of them were resistant to amoxicillin (15/18, 83.4%), tetracycline (15/18 and 83.4%), ceftazidime (14/18 and 77.8%), co-amoxiclav (12/18 and 66.7%), erythromycin (9/18 and 50%), ciprofloxacin (12/18 and 66.7%), and cefotaxime (14/18 and 77.8%). Resistance against imipenem (7/18 and 38.9%) and gentamicin (6/18 and 33.4%) was lower (Figure 1). The rate of MDR *S. sonnei* was 45% and 8/18.

### 3.2. Phage Isolation and Antibacterial Assessment

**Figure 2:**
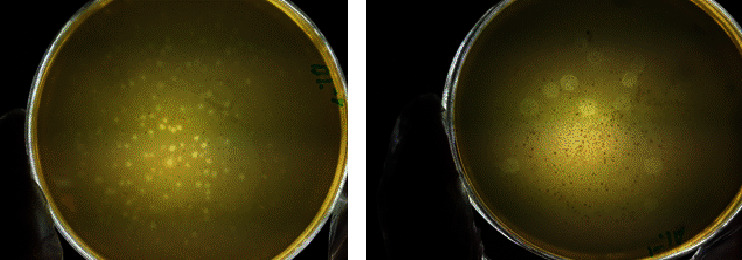
The plaque assay: (a) plaques following phage effect against *S. sonnei*; (b) plaques following phage effect against *S. flexneri.*

Using the spot test, four phages with potential of specific antibacterial effects against *S. sonnei* and *S. flexneri* standard strains were isolated. The double layer agar technique was also performed for the separation of those phages with higher activity. All the phages were from sewage samples (Figure 2).

### 3.3. Phage Morphological Characterization Using TEM

**Figure 3:**
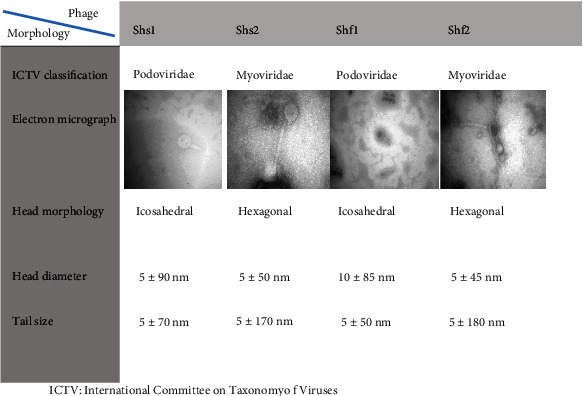
The features of phages isolated in this study.

The TEM analysis depicted that Shf1 and Shf2 phages were different, while Shf2 and Shs2 were similar (Figure 3). Other features of phages have been represented in Figure 3.

In the LB medium, the growth inhibitory effect of phages 4 against *Shigella* spp. outlined that after 24 hrs of coculture, phage cocktail (Shs1, Shs2, Shf1, and Shf2) exerted higher antibacterial activity compared to the single each phage.

### 3.4. Host Specificity

The host specificity of phages was evaluated using spot test and clinical isolates of *S. sonnei* (*n* = 10) and *S. flexneri* (*n* = 8). Totally, 83% isolates were susceptible to cocktail phage (Shs1 + Shs2 + Shf1 + Shf2), while each single phage exerted lower antibacterial effect.

### 3.5. Phage Cocktail Effect against Standard Strains

**Figure 4:**
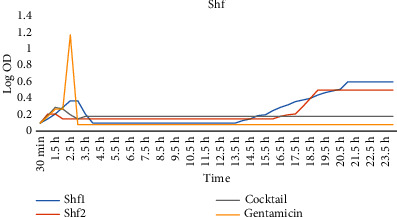
The antibacterial effects of phages against *S. flexneri* in the LB medium.

**Figure 5:**
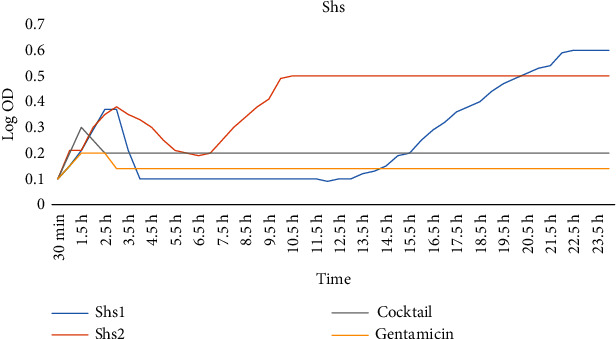
The antibacterial effects of phages against *S. sonnei* in the LB medium.

The antibacterial effect of cocktail phage (Shs1 + Shs2 + Shf1 + Shf2) against *S. sonnei* and *S. flexneri* standard strains in LB medium exhibited stable inhibition compared to that of single phage exposure. Hence, the cocktail conferred significantly higher growth inhibitory effect (Figures 4 and 5).

**Figure 6:**
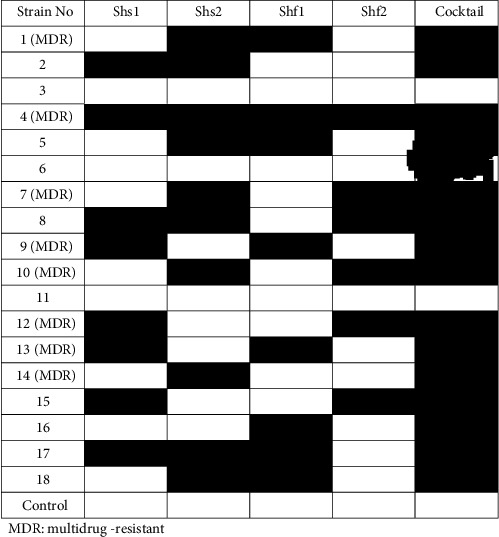
The host/strain specificity of bacteriophages; single phage had narrow activity while cocktail phage exhibited vast antibacterial activity.

We observed that cocktail phage had wider antibacterial activity compared to single phage against *Shigella* spp. strains (Figure 6). Moreover, most of isolates except for two susceptible strains were susceptible to cocktail phage.

## 4. Discussion

In this study, phage cocktail from sewage which included Shs1, Shs2, Shf1, and Shf2 conferred substantial antibacterial effects against *S. sonnei* and *S. flexneri*. However, single phage application effects were unstable. Shs1, Shs2, Shf1, and Shf2 phages belonged to *Podoviridae* and *Myoviridae* families according to the TEM morphological features. Moreover, the phages had host specificity using the spot test performed against clinical isolates.

In a study, lytic bacteriophage isolated from wastewater conferred antibacterial and antibiofilm effects against colistin-resistant *A. baumannii* clinical isolates [21]. In another study, Ghajavand et al. [22] obtained two lytic phages for *Acinetobacter baumannii* from hospital effluents, which had a narrow host range, and both phages significantly reduced the turbidity of *A. baumannii*, indicating potential agents for controlling these species. In an *in vitro* study, the human brain and bladder cell lines were grown in the presence of *A. baumannii* and bacteriophage specific for this bacterium, which significantly reduced lactate dehydrogenase compared to samples without bacteriophage treatment, which highlighted that phages can protect human cells from *A. baumannii* cell invasion.

In an *in vivo* study, a bacteriophage against carbapenem-resistant *A. baumannii* was isolated from wastewater, and using *Galleria mellonella* and a model of mice with acute pneumonia, the antibacterial effect of phages showed no mortality or serious side effects in phage-treated groups, and acceptable results and short-time treatment were achieved in the phage-treated group in two animal samples [23]. In a study comparing antibiotics and phages in the treatment of *Klebsiella* spp. wound infections, phage delineated the highest therapeutic effects at different doses compared to the gentamicin and silver nitrate [24]. In Soleimani et al.'s study, the antibacterial effect of bacteriophage isolated from urban wastewater was evaluated to treat mice with pneumonia caused by *K. pneumoniae* and finally reduced the bacterial count compared to the control group [25].

Similar to our study, phage cocktail against *S. sonnei* and *S. flexneri* inhibited the bacterial growth compared to single form of each phage [8]. In a study in South Korea, a virulent *Myoviridae* bacteriophage, pSs-1 from environmental samples exhibited antibacterial effect against *S. sonnei* and *S. flexneri* and suggested to have the ability to replace antibiotics in the treatment of shigellosis [26].

Bacteriophages have had the ability to reduce *S. flexneri* by 2 logs, which suggests that phages have a high potential to develop an alternative strategy against *S. flexneri* contamination in food [7]. A specific bacteriophage against *S. flexneri* 2457T, and by testing the culture curve and infection assay with HT-29 colorectal adenocarcinoma cells and a human intestinal-derived epithelial monolayer model, was able to eradicate *S. flexneri* 2457T. The phage also prevented the attachment and invasion of *S. flexneri* 2457T to epithelial cells in both models of infection [5]. Employment of cocktail forms of phages has conferred substantial antibacterial effects compared to single forms, though more in-depth studies are required [27, 28]. Previous studies have unraveled that phages can be proper to hinder and control bacterial pathogens [5, 7–27]. Major limitations of our study included low number of clinical isolates, lack of performance of phage stability tests in various temperatures and pH, lack of biofilm formation and antibiofilm assessment of phages, and sequencing of phages.

## 5. Conclusion

In this study, phage cocktail from sewage which included Shs1, Shs2, Shf1, and Shf2 conferred substantial and host-specific antibacterial effects against *S. sonnei* and *S. flexneri*. However, single phage application effects were unstable. Shs1, Shs2, Shf1, and Shf2 phages belonged to *Podoviridae* and *Myoviridae* families according to TEM morphological features. Moreover, the phages had host specificity using the spot test performed against clinical isolates.

## Data Availability

The data will be available by the request from the corresponding author.

## References

[1] van den Beld M. J., Warmelink E., Friedrich A. W. (2019). Incidence, clinical implications and impact on public health of infections with *Shigella spp*. and entero-invasive *Escherichia coli* (EIEC): results of a multicenter cross-sectional study in the Netherlands during 2016–2017. *BMC Infectious Diseases*.

[2] Wang Y., Ma Q., Hao R. (2019). Antimicrobial resistance and genetic characterization of *Shigella spp*. in Shanxi Province, China, during 2006–2016. *BMC Microbiology*.

[3] Seferbekova Z., Zabelkin A., Yakovleva Y. (2021). High rates of genome rearrangements and pathogenicity of *Shigella spp*. *Frontiers in Microbiology*.

[4] Ranjbar R., Farahani A. (2019). *Shigella*: antibiotic-resistance mechanisms and new horizons for treatment. *Infection and Drug Resistance*.

[5] Llanos-Chea A., Citorik R. J., Nickerson K. P. (2019). Bacteriophage therapy testing against *Shigella flexneri* in a novel human intestinal organoid-derived infection model. *Journal of Pediatric Gastroenterology and Nutrition*.

[6] Shahin K., Bao H., Komijani M. (2019). Isolation, characterization, and PCR-based molecular identification of a *siphoviridae* phage infecting *Shigella dysenteriae*. *Microbial Pathogenesis*.

[7] Shahin K., Bouzari M. (2018). Bacteriophage application for biocontrolling Shigella flexneri in contaminated foods. *Journal of Food Science and Technology*.

[8] Shahin K., Bouzari M., Komijani M., Wang R. (2020). A new phage cocktail against multidrug, ESBL-producer isolates of *Shigella sonnei* and *Shigella flexneriwith* highly efficient bacteriolytic activity. *Microbial Drug Resistance*.

[9] Shahin K., Zhang L., Delfan A. S. (2021). Effective control of *Shigella* contamination in different foods using a novel six-phage cocktail. *LWT*.

[10] Islam M., Zhou Y., Liang L. (2019). Application of a phage cocktail for control of *Salmonella* in foods and reducing biofilms. *Viruses*.

[11] Lewis R., Hill C. (2020). Overcoming barriers to phage application in food and feed. *Current Opinion in Biotechnology*.

[12] Cha Y., Son B., Ryu S. (2019). Effective removal of staphylococcal biofilms on various food contact surfaces by *Staphylococcus aureus* phage endolysin LysCSA13. *Food Microbiology*.

[13] de Melo A. G., Levesque S., Moineau S. (2018). Phages as friends and enemies in food processing. *Current Opinion in Biotechnology*.

[14] Sommer J., Trautner C., Witte A. K. (2019). Don’t shut the stable door after the phage has bolted—the importance of bacteriophage inactivation in food environments. *Viruses*.

[15] Cristobal-Cueto P., García-Quintanilla A., Esteban J., García-Quintanilla M. (2021). Phages in food industry biocontrol and bioremediation. *Antibiotics*.

[16] Clinical and Laboratory Standards Institute (2019). *Performance Standards for Antimicrobial Susceptibility Testing. CLSI Supplement M100*.

[17] Khan Mirzaei M., Nilsson A. S. (2015). Isolation of phages for phage therapy: a comparison of spot tests and efficiency of plating analyses for determination of host range and efficacy. *PLoS One*.

[18] Aghaee B. L., Khan Mirzaei M., Alikhani M. Y., Mojtahedi A., Maurice C. F. (2021). Improving the inhibitory effect of phages against Pseudomonas aeruginosa isolated from a burn patient using a combination of phages and antibiotics. *Viruses*.

[19] Pelyuntha W., Ngasaman R., Yingkajorn M., Chukiatsiri K., Benjakul S., Vongkamjan K. (2021). Isolation and characterization of potential *Salmonella* phages targeting multidrug-resistant and major serovars of *Salmonella* derived from broiler production chain in Thailand. *Frontiers in Microbiology*.

[20] Petsong K., Benjakul S., Chaturongakul S., Switt A. I. M., Vongkamjan K. (2019). Lysis profiles of *Salmonella* phages on *Salmonella* isolates from various sources and efficiency of a phage cocktail against S. Enteritidis and S. Typhimurium. *Microorganisms*.

[21] Ebrahimi S., Sisakhtpour B., Mirzaei A., Karbasizadeh V., Moghim S. (2021). Efficacy of isolated bacteriophage against biofilm embedded colistin-resistant *Acinetobacter baumannii*. *Gene Reports*.

[22] Ghajavand H., Esfahani B. N., Havaei A., Fazeli H., Jafari R., Moghim S. (2017). Isolation of bacteriophages against multidrug resistant *Acinetobacter baumannii*. *Research in Pharmaceutical Sciences*.

[23] Jeon J., Park J.-H., Yong D. (2019). Efficacy of bacteriophage treatment against carbapenem-resistant *Acinetobacter baumannii* in *Galleria mellonella* larvae and a mouse model of acute pneumonia. *BMC Microbiology*.

[24] Bull J., Vimr E., Molineux I. (2010). A tale of tails: sialidase is key to success in a model of phage therapy against K1-capsulated *Escherichia coli*. *Virology*.

[25] Zurabov F., Zhilenkov E. (2021). Characterization of four virulent Klebsiella pneumoniae bacteriophages, and evaluation of their potential use in complex phage preparation. *Virology Journal*.

[26] Jun J. W., Giri S. S., Kim H. J. (2016). Bacteriophage application to control the contaminated water with *Shigella*. *Scientific Reports*.

[27] Tanji Y., Shimada T., Fukudomi H., Miyanaga K., Nakai Y., Unno H. (2005). Therapeutic use of phage cocktail for controlling *Escherichia coli* O157:H7 in gastrointestinal tract of mice. *Journal of Bioscience and Bioengineering*.

[28] Mendes J. J., Leandro C., Mottola C. (2014). In *vitro* design of a novel lytic bacteriophage cocktail with therapeutic potential against organisms causing diabetic foot infections. *Journal of Medical Microbiology*.

